# Role of Neutrophils in Cardiac Injury and Repair Following Myocardial Infarction

**DOI:** 10.3390/cells10071676

**Published:** 2021-07-02

**Authors:** Yonggang Ma

**Affiliations:** Department of Molecular Pharmacology and Physiology, Morsani College of Medicine, University of South Florida, Tampa, FL 33612, USA; yma4@usf.edu; Tel.: +1-813-974-1516

**Keywords:** adaptive immunity, angiogenesis, cardiac remodeling, inflammation, macrophage, myocardial infarction, neutrophil

## Abstract

Neutrophils are first-line responders of the innate immune system. Following myocardial infarction (MI), neutrophils are quickly recruited to the ischemic region, where they initiate the inflammatory response, aiming at cleaning up dead cell debris. However, excessive accumulation and/or delayed removal of neutrophils are deleterious. Neutrophils can promote myocardial injury by releasing reactive oxygen species, granular components, and pro-inflammatory mediators. More recent studies have revealed that neutrophils are able to form extracellular traps (NETs) and produce extracellular vesicles (EVs) to aggravate inflammation and cardiac injury. On the contrary, there is growing evidence showing that neutrophils also exert anti-inflammatory, pro-angiogenic, and pro-reparative effects, thus facilitating inflammation resolution and cardiac repair. In this review, we summarize the current knowledge on neutrophils’ detrimental roles, highlighting the role of recently recognized NETs and EVs, followed by a discussion of their beneficial effects and molecular mechanisms in post-MI cardiac remodeling. In addition, emerging concepts about neutrophil diversity and their modulation of adaptive immunity are discussed.

## 1. Introduction

Neutrophils are the most abundant circulating leukocytes in humans and act as the first responders to infection and sterile inflammation. Due to their limited life span and terminal differentiation, the bone marrow continuously produces neutrophils through granulopoiesis to maintain homeostasis [1]. During infection or injury, the bone marrow produces more neutrophils through emergency or reactive granulopoiesis to meet the high demand of the host [1]. In addition, the spleen also generates neutrophils in severe conditions through a process known as extramedullary hematopoiesis [2]. CXCL12-CXCR4 signaling is a retention signal that prevents neutrophil egress from the bone marrow, while the CXCL1/2-CXCR2 signal drives their mobilization into the peripheral blood [3,4,5]. Traditional views are that naïve tissues are believed to be free of neutrophils. However, a recent study using neutrophil reporter mice showed that in the steady state, neutrophils actively infiltrate most tissues, including the heart [6]. Their lifespan in most tissues is one day or less [7]. Similar to resident macrophages, tissue neutrophils adopt features tailored to the needs of those tissues, and support organ homeostasis [7]. Following myocardial infarction (MI), CXCL12-CXCR4 signaling is disrupted [8], which allows neutrophil mobilization to the peripheral blood, leading to neutrophilia.

Blood neutrophils infiltrate the ischemic myocardium in large quantities, within a few hours after MI onset [9,10]. They are attracted by cell debris and inflammatory mediators released by activated resident cells. MI induces cardiac cell damage, which leads to the release of an array of heterogenous molecules, including damage-associated molecular patterns (DAMPs) and alarmins. Cardiac-resident macrophages and the endothelium detect these danger signals, initiating neutrophil recruitment [11]. Neutrophils express a wide range of receptors, including pattern recognition receptors (e.g., toll-like receptors) and receptors for chemokines, cytokines, and adhesion molecules. These receptors allow for their recognition of and response to distinct DAMPs, alarmins, chemokines, or cytokines released in the ischemic heart [11,12]. Neutrophil extravasation from microvessels depends on the interaction of integrins and adhesion molecules expressed on neutrophils and endothelial cells [13]. Once recruited into the ischemic myocardium, activated neutrophils exert a plethora of biological functions.

Recruited neutrophils initially aim to phagocytose and clear dead cell debris caused by ischemia. However, they concomitantly cause collateral cardiac injury by releasing reactive oxygen species (ROS), proteolytic enzymes, and inflammatory mediators [14,15]. In addition, neutrophils are able to form extracellular traps (NETs) and release extracellular vesicles (EVs) that contain a multitude of inflammatory mediators. Clinically, high peripheral neutrophil counts are associated with adverse outcomes and high mortality in patients with coronary syndromes [16,17]. On the contrary, accumulating evidence shows that neutrophils also have anti-inflammatory, pro-angiogenic, and pro-reparative effects, thus being beneficial for cardiac wound healing [10,11,18]. This review summarizes the current knowledge on neutrophils’ deleterious effects with a focus on the role of recently recognized NETs and EVs, followed by a discussion of their pro-reparative roles and molecular mechanisms. In addition, we discuss the emerging concept about neutrophil diversity and their regulation of an adaptive immune response.

## 2. Neutrophil-Mediated Cardiac Injury

Traditionally, neutrophils are considered mostly, if not completely, detrimental in the setting of acute MI. This idea is supported by both clinical and experimental studies. The circulating neutrophil count positively correlates to infarct size, death, and heart failure development [19,20]. Experimental studies have also revealed that either neutrophil depletion or inhibition reduces cardiac injury and infarct size [21,22,23,24].

### 2.1. Neutrophil Respiratory Burst, Degranulation, Secretion of Inflammatory Mediators, and No-Reflow Induced by Neutrophils

Neutrophils possess a number of weapons to defend against a challenge or cause collateral tissue injury. Through a respiratory burst, neutrophils generate large amounts of ROS in a nicotinamide adenine dinucleotide phosphate (NADPH)-dependent manner. ROS can directly cause tissue injury by modifying amino acids, proteins, and lipids [14,25]. ROS also stimulate the release of pro-inflammatory factors in the ischemic myocardium [26]. Upon degranulation, neutrophils release a wide range of pre-synthesized granular proteins, including myeloperoxidase (MPO), serine proteases, and matrix metalloproteinases (MMPs). These enzymes can cause myocyte death and ECM degradation and have been shown to be detrimental in MI-induced cardiac remodeling [14]. In addition, neutrophils can secrete cytokines (e.g., tumor necrosis factor (TNF)-α, interleukin (IL)-1β, and IL-8) and chemokines (CXCL1, 2, 3, and 8) [13,27], which increase inflammation and negatively affect myocyte contractility [28]. 

**Figure 1:**
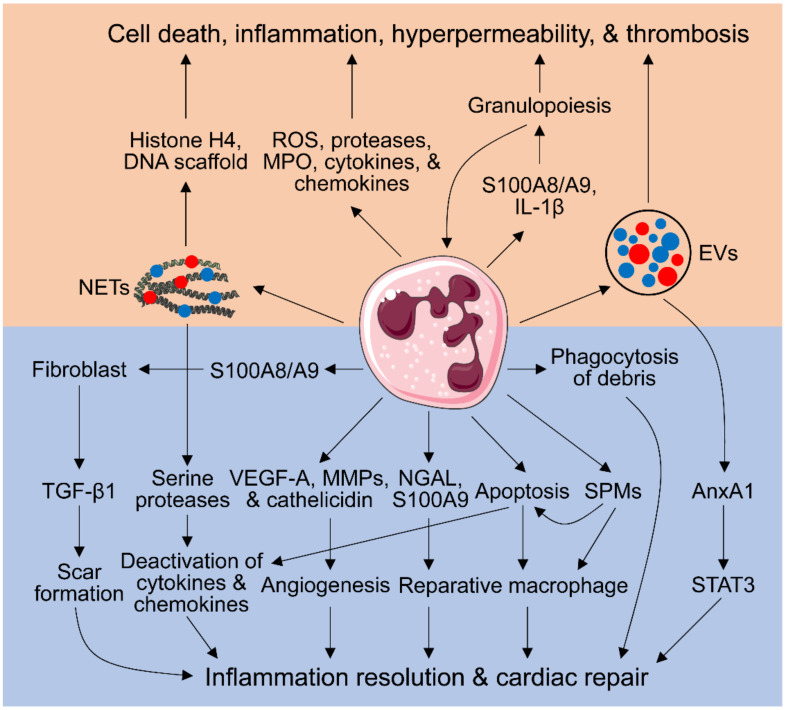
Detrimental and beneficial roles of neutrophils in myocardial infarction (MI) wound healing. It is well known that neutrophils can promote myocardial injury by releasing reactive oxygen species (ROS), granular components, and pro-inflammatory mediators. Recent studies show that neutrophils are able to form extracellular traps (NETs) and produce extracellular vesicles (EVs) to increase inflammation and cardiac injury. In addition, neutrophils enhance granulopoiesis, forming a positive feed-forward loop for neutrophil production and acute inflammation. On the contrary, emerging evidence reveals that neutrophils are indispensable for appropriate wound healing after MI. They promote inflammation resolution, angiogenesis, and scar formation by generating a wide array of pro-reparative factors, including neutrophil gelatinase-associated lipocalin (NGAL), vascular endothelial growth factor-A (VEGF-A), cathelicidin, annexin A1 (AnxA1), and specialized pro-resolving mediators (SPMs). MPO, myeloperoxidase; MMPs, matrix metalloproteinases. The red and blue dots represent different proteins or other molecules. Images of cells are from Servier Medical ART (Accessed date 1 June 2021 https://smart.servier.com).

No-reflow occurs after reperfusion of an infarcted artery in the setting of MI and is mainly caused by the obstruction of myocardial microcirculation. Patients with no-reflow have a worse prognosis and higher mortality [29]. Endothelial cellular swelling and protrusions, cardiomyocyte swelling, tissue edema, vasospasm, and microvascular thrombosis contribute to microvascular obstruction [30]. In addition, activated neutrophils exhibit decreased deformability and can cause microvascular plugging and no-reflow after ischemia/reperfusion (I/R) [31,32]. These deleterious effects of neutrophils in MI and I/R have been well established and extensively discussed in several reviews (Figure 1) [14,26,31].

### 2.2. Neutrophil Extracellular Traps (NETs)

NETs are chromatin filaments fused with granular and cytoplasmic components. NET release mainly occurs through a cell death process referred to as suicidal or lytic NETosis [33,34]. This process is accompanied by the permeabilization of the nuclear envelop and plasma membrane rupture, causing neutrophil death. An alternative mechanism is termed vital or non-lytic NET extrusion, which leads to the rapid release of NETs in the absence of cell death [34]. Peptidylarginine deiminase 4 (PAD4) plays a critical role in NET formation. PAD4 converts positively charged arginyl residues on chromatin histones to citrulline, which lacks a charge. This reaction releases the ionic bonds that mediate the tight association of negatively charged DNA with histones in the nucleosomes, causing DNA to unfurl and chromatin decondensation [35]. After decoration with cytoplasmic components, the decondensed chromatin is released into extracellular space, thereby forming NETs [13].

Although NETs were originally identified as a host defense mechanism trapping pathogens [36], a body of evidence has revealed that they also play a detrimental role in sepsis, autoimmune disease, cancer, thrombosis, and cardiovascular disease [37,38]. They are detected in culprit arteries of acute MI patients. Plasma thrombin is reported to be responsible for NET generation through activating platelets [39]. NETs contribute to thrombosis by facilitating fibrin deposition and the formation of a fibrin network [40], implying that NETs may be involved in MI occurrence. NET-mediated microthrombosis also contributes to myocardial no-reflow after I/R [41]. NETs can activate macrophage NLRP3 inflammasome to release IL-1β and IL-18 [42,43]; in turn, IL-18 stimulates NET release [42], forming a vicious pro-inflammatory cycle. More importantly, NETs positively correlate with the occurrence of adverse cardiac events, worse ST segment resolution, infarct size, and cardiac dysfunction in patients with MI [44,45,46,47]. Either inhibition of NET generation by PAD4 deficiency or degradation of NETs by DNase I has been shown to protect from myocardial I/R injury, evidenced by smaller infarct size, less neutrophil infiltration, and improved cardiac function [48]. Likewise, pharmacological inhibition of PAD4 activity reduces MI-induced NET formation, inflammatory reaction, and cardiomyocyte apoptosis, thereby improving cardiac function [49]. Mechanistically, histone H4 within NETs is shown to induce lytic cell death [50]. It has also been reported that the DNA scaffold of NETs is required for tissue factor to activate the coagulation cascade (Figure 1) [39]. Whether the other functions of NETs need the structure integrity remains largely unknown.

On the contrary, NETs exhibit anti-inflammatory features. Serine proteases within NETs can degrade cytokines and chemokines (Figure 1), thus blunting inflammation [51,52]. In neutrophilic inflammation, NET deficiency exacerbates the inflammatory response, which is alleviated by the adoptive transfer of aggregated NETs [51]. This is associated with the capability of NETs to promote macrophage polarization toward a reparative phenotype [53,54]. Serine proteases, key components of NETs, have been shown to be detrimental in MI but beneficial in gout [14,51], indicating that NETs’ role is context-dependent. As the composition of NETs varies depending on the stimulus, the dual role of NETs is also component-dependent.

### 2.3. Extracellular Vesicles (EVs)

EVs are a heterogeneous collection of membranous vesicles released by a wide array of cells. Based on their size and the pathways involved in their production, EVs are classified into three groups: exosomes (≤100–150 nm), microvesicles (MVs; up to 1000 nm), and apoptotic bodies (>1000 nm) [13,55]. They take part in cellular crosstalk by engaging receptors on the cell surface or by delivering EV cargo into the target cell [56]. In spite of being present in small amounts in the steady state, neutrophil-derived MVs are profoundly elevated in inflammatory conditions, both in the peripheral blood and at sites of tissue inflammation [57,58]. Neutrophil EVs can stimulate endothelial cell production of inflammatory mediators (IL-6, monocyte chemoattractant protein-1, and tissue factor) [59,60] and increase endothelial microvascular permeability (Figure 1) [61], both of which contribute to acute inflammation. 

On the contrary, neutrophil-derived EVs show protective effects. Administration of neutrophil MVs carrying annexin A1 (AnxA1) inhibits inflammation, an effect that disappears when injecting MVs devoid of AnxA1 [62]. This indicates that AnxA1 is responsible for the anti-inflammatory feature of neutrophil EVs. Similarly, intra-articular injection of AnxA1^+^ MVs alleviates arthritis-induced cartilage degradation, which is associated with increased transforming growth factor (TGF)-β1 generation, leading to cartilage protection [63]. In myocardial I/R, AnxA1 overexpression inhibits neutrophil infiltration by activating the STAT3 signaling pathway (Figure 1) [64]. In addition, neutrophil MVs prevent inflammatory activation of macrophages [65]. The distinct pro-inflammatory and pro-resolving effects of neutrophil EVs depend on their cargo composition, which varies based on the stimulus utilized for their generation and the neutrophil status during EV production [66,67]. However, the role of neutrophil-derived EVs in MI remains to be defined.

### 2.4. Aggravating Granulopoiesis by Neutrophils

In steady-state conditions, neutrophil production is tightly regulated by granulocyte colony stimulating factor (G-CSF), a cytokine primarily secreted by immune cells, including neutrophils [68]. MI enhances granulopoiesis, resulting in the increased production of neutrophils in the bone marrow [1,69]. Following infection or sterile inflammation (e.g., MI), granulopoiesis can be enhanced by DAMPs and inflammatory cytokines (e.g., IL-6, IL-3, and granulocyte-macrophage colony-stimulating factor) [1,70]. A seminal study shows that neutrophils can induce granulopoiesis [71]. Neutrophils recruited to the infarcted heart release alarmins S100A8/A9 heterodimer, which stimulate IL-1β secretion by neutrophils. The released IL-1β, delivered through the blood circulation, binds with its receptor on hematopoietic stem and progenitor cells in the bone marrow and stimulates granulopoiesis (Figure 1) [71]. Thus, neutrophils enhance granulopoiesis, forming a positive feed-forward loop for neutrophil production. More importantly, disruption of S100A8/A9 and downstream signaling cascade inhibit MI-induced granulopoiesis and alleviate cardiac dysfunction [71]. This is in line with previous work revealing that S100A8/A9 blockade reduces neutrophil production and infiltration into the myocardium, as well as improves cardiac function after MI [72,73]. 

## 3. Neutrophil-Dependent Myocardial Repair

In acute infection or inflammation, neutrophils are not only essential for the removal of pathogens or cell debris, but also for the resolution of inflammation and return to homeostasis [10,74]. Emerging evidence shows that neutrophils are required for appropriate wound healing post-MI. The section below discusses the pro-reparative roles of neutrophils in MI, as well as in infection or sterile inflammation if data on MI are not available.

### 3.1. Phagocytosis of Tissue and Cellular Debris by Neutrophils

As professional phagocytes, neutrophils are involved in removing necrotic myocardium and cellular debris. This process relies on neutrophil spreading, a process involving increasing the size of the neutrophil cell membrane [75]. Neutrophil phagocytosis is initiated by adhesion of neutrophil integrins to cellular debris, which results in an increase in intracellular calcium and calpain activation. Calpain activation induces its translocation from the cytosol to the cell membrane and aids in the formation of the phagocytic cup [76]. In addition, calpain cleavage of p81 creates space between the F-actin protrusions and the F-actin membrane, which increases the neutrophil membrane size and allows the neutrophil to engulf the debris [75]. Ganoderma lucidum, a Chinese medical fungus, is effective in the treatment of hypertension, hyperglycemia, neoplasia, and chronic liver disease. These protective effects are at least partially mediated by polysaccharides purified from Ganoderma lucidum (PS-G), which enhance neutrophil phagocytosis [77]. It would be interesting to know whether PS-G administration could improve cardiac wound healing after MI by increasing the phagocytotic capability of neutrophils.

### 3.2. Inflammation Resolution Promoted by Apoptotic Neutrophils

After fulfilling its roles, neutrophils have to be removed in a timely fashion through apoptosis or another mode of death. Delayed neutrophil apoptosis occurs in multiple human inflammatory diseases, including acute coronary syndromes [78,79]. Persistence of neutrophils can cause tissue damage and chronic inflammation. As opposed to necrosis, which releases intracellular components and induces an acute inflammatory response, neutrophil apoptosis exposes phosphatidylserine on the cell outer surface, which signals macrophage efferocytosis (Figure 1) [78]. Tissue neutrophils are mainly removed by macrophages and, to a small extent, by dendritic cells (DCs), exodus to draining lymph nodes [80], or even reverse transendothelial migration back into the vasculature [81,82]. Removal of apoptotic neutrophils initiates the process of inflammation resolution [83]. Phagocytosis of apoptotic neutrophils by macrophages, a process known as efferocytosis, stimulates the production of anti-inflammatory and pro-resolving mediators, including TGF-β1, IL-10, vascular endothelial growth factor (VEGF), and specialized pro-resolving mediators (SPMs) [84,85,86,87], contributing to inflammation resolution and cardiac repair. Pro-resolving lipids and proteins, such as lipoxin A4, resolvin E1, and AnxA1, can induce neutrophil apoptosis and promote their removal by efferocytosis [88]. Dying neutrophils are able to release antimicrobial α-defensins, which increases the phagocytic capacity of macrophages and dampens their release of inflammatory factors [89]. Following MI, MMP-12 inhibition has been shown to suppress neutrophil apoptosis, leading to delayed inflammation resolution and maladaptive remodeling [90]. Inhibition of the macrophage efferocytosis of apoptotic neutrophils or cardiomyocytes by recombinant CXCL4 infusion enhances post-MI cardiac dilation and mortality [91].

In addition, apoptotic neutrophils can scavenge chemokines and cytokines (Figure 1). Apoptotic neutrophils can bind to chemokines and cytokines, without generating biological effects. This precludes them from binding to viable cells [92]. For instance, aspirin-triggered SPMs increase CCR5 expression on apoptotic human neutrophils, which sequesters soluble CCL3 and CCL5 by acting as a decoy receptor [93].

NETosis represents another form of neutrophil death. NETotic neutrophils can also be cleared by macrophages. Macrophages are able to engulf NETs in a cytochalasin D-dependent manner, implying that this is an active, endocytic process [94]. Upon internalization, macrophage degradation of NETs is dependent on TREX1 (DNaseIII) [95]. Similar to the efferocytosis of apoptotic neutrophils, the macrophage uptake of NETs does not induce an inflammatory response [94]. Therefore, this may represent another novel mechanism whereby macrophages promote inflammation resolution. Future studies are needed to decipher whether macrophage removal of NETs contributes to favorable cardiac repair post-MI.

### 3.3. Inducing a Pro-Reparative Macrophage Phenotype by Neutrophils

In general, infarct macrophages exhibit a pro-inflammatory phenotype early (days 1–3) and become polarized toward a pro-reparative subtype later (after day 3) post-MI [96]. One study reported that co-culture of neutrophils with activated macrophages induces a decrease in the pro-inflammatory factors released by macrophages through suppressing nuclear factor-κB activation [97], supporting the concept that neutrophils are capable of modulating macrophage phenotype. Infarct macrophages in neutrophil-depleted animals exhibit lower MerTK expression [98], a receptor that mediates the clearance of apoptotic cells [99]. This indicates that neutrophils polarize macrophages toward a reparative phenotype post-MI. Accordingly, neutrophil depletion results in the accumulation of apoptotic cells, increased fibrosis, and worse cardiac function [98]. Further analysis reveals that neutrophil gelatinase-associated lipocalin (NGAL) mediates the pro-reparative roles of neutrophils as NGAL administration restores macrophage phenotype in neutrophil-depleted mice (Figure 1) [98]. 

In addition to pro-inflammatory effects, S100A9 exhibits pro-reparative roles. Short-term (three days) S100A9 blockade shows beneficial effects [72], while long-term (21 days) blockade adversely impacts myocardial repair and function [100]. Similarly, S100A8/A9 suppresses inflammation in rat autoimmune myocarditis by inhibiting cytokine production [101]. Mechanistically, S100A9 promotes the transition from inflammatory monocytes to reparatory Ly6C^lo^MerTK^hi^ macrophages by upregulating the transcription factor Nur77 (Figure 1), thus promoting the clearance of dead cells and debris [100]. In summary, S100A9 stimulates myeloid cell generation and trafficking to the ischemic heart at acute phase (three days) post-MI, but promotes reparatory macrophage production after the acute inflammatory period. Long-term S100A9 blockade closely recapitulates the negative effects of neutrophil depletion on post-MI cardiac recovery [98]. 

### 3.4. Pro-Angiogenic Neutrophils

Angiogenesis is an integral component of optimal would healing after MI. Newly formed vessels can provide nutrients and oxygen to the tissue around the infarct border region, limiting infarct expansion. Strategies that induce angiogenesis have been shown to improve post-MI cardiac repair and function [102,103]. Neutrophils have long been known to release VEGF-A, the major stimulator of angiogenesis [104]. Adenosine released by neutrophils can induce macrophage production of VEGF [105]. Circulating CXCR4^hi^ neutrophils recruited by VEGF-A release a large amount of MMP-9 [106], and MMP-9 can induce angiogenesis by degrading ECM to release matrix-bound VEGF-A and generate pro-angiogenic ECM fragments [18,107]. Interestingly, MMP-9 deletion facilitates angiogenesis following MI [108], indicating that MMP-9 also displays antiangiogenic roles. A recent study identified a blood pro-angiogenic subset of neutrophils in humans and mice that are CD49d^+^VEGFR1^hi^CXCR4^hi^, and inhibiting their recruitment impairs vessel neoformation in a transplantation-based angiogenesis model [109]. In a mouse model of transplanting pancreatic islets into the cremaster muscles, neutrophils migrate in a directional manner to angiogenic hotspots around the islet, where endothelial sprouting occurs [110]. More importantly, neutrophil depletion inhibits vessel growth. In a mouse model of artery injury, neutrophil-borne cathelicidin (mouse CRAMP and human LL-37) facilitates reendothelization and limits neointima formation after stent implantation, thus reducing stenosis [111]. Whether cathelicidin also promotes post-MI angiogenesis and cardiac wound healing needs to be investigated. 

Neutrophils also promote angiogenesis indirectly. Neutrophils are the primary source of AnxA1 in the infarcted heart [112]. AnxA1 facilitates macrophage polarization toward a pro-angiogenic phenotype, which favors angiogenesis by secreting VEGF-A [112].

### 3.5. Neutrophil Generation of Specialized Pro-Resolving Mediators (SPMs)

SPMs are derived from essential fatty acids, including arachidonic acid (AA; C20:n-6), eicosapentaenoic acid (EPA; C20:n-3), and docosahexaenoic acid (DHA; C22:n-3) in a lipoxygenase (LOX)-dependent manner [113]. The major SPM families consist of lipoxins from AA, E-series resolvins from EPA, as well as D-series resolvins, protectins, and maresins from DHA. SPMs exert their biological functions by activating corresponding receptors. For example, the lipoxin A4 receptor ALX, also known as FPR2, binds LXA4 and 15-epi-LCA4 to orchestrate the resolution of inflammation [113]. Other high-affinity receptors have also been identified: CMKLR1 and CHEMR23 for resolvin E1 [114], GPR32 and ALX for resolvin D1 [115], as well as GPR18 for resolvin D2 [116].

SPMs have essential roles in facilitating the resolution of inflammation. They can limit neutrophil recruitment, counter-regulate pro-inflammatory cytokines, and facilitate macrophage phagocytosis (Figure 1) [113]. SPMs are also able to enhance neutrophil-mediated bacterial clearance and permit neutrophil apoptosis to take place (Figure 1) [117]. Defects in SPM pathways contribute to the development of unresolved chronic inflammation. Neutrophils are able to produce lipoxin A4, resolvin D1, and 13-series resolvins in a 5-LOX dependent manner [118,119]. Moreover, activated neutrophils highly express ALX/FPR2 [118,120]. Pharmacological inhibition of FPR2 disturbs leukocyte recruitment and elicits non-resolving inflammation following MI [120]. However, the relative contribution of neutrophil-derived SPMs to inflammation resolution in an MI setting is largely uninvestigated.

### 3.6. Regulation of Fibroblast Functions by Neutrophils

In response to MI, cardiac fibroblasts differentiate into myofibroblasts, which secrete collagens and other ECM proteins to form a scar [121]. Insufficient scar formation contributes to cardiac rupture and adverse remodeling post-MI [122], while excess ECM deposition in the non-infarct remote region can cause cardiac fibrosis [121]. TGF-β1 is the master cytokine that regulates scar formation. TGF-β1 is mainly produced by fibroblasts, macrophages, and T cells in the heart [123]. Neutrophils have been shown to upregulate TGF-β1 expression by fibroblasts (Figure 1). In vitro, co-culture of naïve neutrophils with cardiac fibroblasts upregulates TGF-β1 expression [124]. NETs have also been shown to upregulate TGF-β1 expression and collagen production by fibroblasts and to increase their proliferation and migration [53,125]. Depletion of neutrophils in vivo prevents TGF-β1 upregulation post-MI [124]. Neutrophil-derived S100A8/A9 activates cardiac fibroblasts in angiotensin II infusion induced hypertension [126], implying that S100A8/A9 may mediate neutrophil-induced TGF-β1 upregulation by fibroblasts.

## 4. Neutrophil Heterogeneity and Plasticity

**Figure 2:**
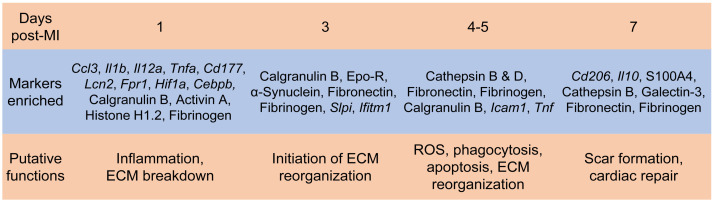
Neutrophils in the infarcted myocardium at different time points post-MI exhibit distinct phenotypes and functions.

Neutrophils are historically considered a homogenous population of cells with highly conserved functions. However, accumulating evidence over the past decade shows phenotypic heterogeneity of blood neutrophils in homeostasis and tissue neutrophils after infection or injury. Distinct subsets of neutrophils in the steady state, infection, and sterile inflammation have been reviewed elsewhere [127,128,129]. We focus here on neutrophils in the MI heart. We previously showed the existence of N1 (Ly6G^+^CD206^-^) and N2 (Ly6G^+^CD206^+^) neutrophil phenotypes in the MI heart [130]. N1 is pro-inflammatory with high expression of pro-inflammatory markers (*Ccl3*, *Il1b*, *Il12a*, and *Tnfα*), while N2 expresses high levels of anti-inflammatory *Cd206* and *Il10* (Figure 2). Although N1 neutrophils are always predominant (>80% of total neutrophils at each time point), the percentage of N2 neutrophils increases post-MI, from 2.4% at day 1 to 18.1% at day 7. In vitro, N1 and N2 phenotypes can be induced by interferon-γ+ lipopolysaccharide or IL-4, respectively. Correlation analysis further reveals that N1 is positively associated with infarct wall thinning, probably due to higher generation of MMP-12 and MMP-25. The peripheral blood does not contain CD206^+^ N2 neutrophils, indicating that N2 is formed locally in the ischemic heart microenvironment. Exogenous administration of IL-4 after MI reduces the expression of pro-inflammatory cytokines in neutrophils [131], implying the inhibition of the N1 phenotype. Ly6G^hi^CXCR2^+^ and Ly6G^lo^CCR2^+^ neutrophil subsets have also been identified in the blood and MI heart [73]. Infiltration of CXCR2^+^ neutrophils peaks at 12 h post-I/R, returning to baseline levels at day 7. In contrast, recruitment of CCR2^+^ cells peaks at day 3 and remains elevated at day 7 after I/R [73]. N1 vs. CXCR2^+^ and N2 vs. CCR2^+^ neutrophils appear to temporally coincide in the ischemic heart. It would be interesting to know whether they represent the same type of neutrophils.

Using an aptamer proteomics approach, we identified cardiac neutrophil proteome shift over the first week after MI [132]. Day 1 cardiac neutrophils exhibited a high degranulation with increased MMP activity. D3 neutrophil profiles showed upregulation of apoptosis and induction of ECM organization. D5 neutrophils further increased their ECM reorganization profile, and D7 neutrophils display a reparative signature (Figure 2). More recently, using single-cell RNA sequencing combined with cell surface epitope detection, six different clusters with specific time-dependent patterning and proportions were identified in cardiac neutrophils from days 1, 3, and 5 post-MI mice [133]. Day 1 neutrophils were characterized by a gene expression pattern similar to bone marrow neutrophils (*Cd177*, *Lcn2*, and *Fpr1*) and putative activity of the transcriptional regulators involved in the hypoxic response (*Hif1a*) and emergency granulopoiesis (*Cebpb*). In contrast, days 3 and 5 neutrophils exhibited two major subsets: SiglecF^hi^ vs. SiglecF^lo^ phenotypes. SiglecF^hi^ neutrophils accounted for approximately 25% of cardiac neutrophils at day 1 and represented more than 50% of neutrophils at day 4 post-MI [134]. The SiglecF^hi^ subtype was enriched for *Icam1* and *Tnf* and displayed enhanced effector functions (e.g., phagocytosis and ROS production), while SiglecF^lo^ was abundant in *Slpi* and *Ifitm1* expression (Figure 2). SiglecF has been shown to induce apoptosis in eosinophils [135]. Since SiglecF upregulation on neutrophils coincides with the inflammation resolution phase, it has been proposed that SiglecF upregulation on neutrophils may induce its apoptosis, which contributes to macrophage efferocytosis and subsequent resolution of inflammation. Future studies are warranted to determine the functional consequences of neutrophil temporal heterogeneity in post-MI cardiac remodeling. 

## 5. Neutrophils and Adaptive Immunity

Increasing evidence suggests that neutrophils modulate an adaptive immune response. Under normal conditions, a small but persistent population of neutrophils is present in the parenchyma of lymph nodes [136,137]. Following infection, blood neutrophils rapidly traffic into lymph nodes across high endothelial venules (HEVs) [137]. This process is mediated by the ligation of L-selectin and P-selectin glycoprotein ligand-1 on neutrophils with peripheral node addressin (PNAd) on HEVs and P-selectin on platelets [137,138]. In addition, neutrophils recruited to inflamed tissue can cross lymphatic vessels, thereby entering lymph nodes, which is dependent on CD11b and CXCR4 [139,140]. 

**Figure 3:**
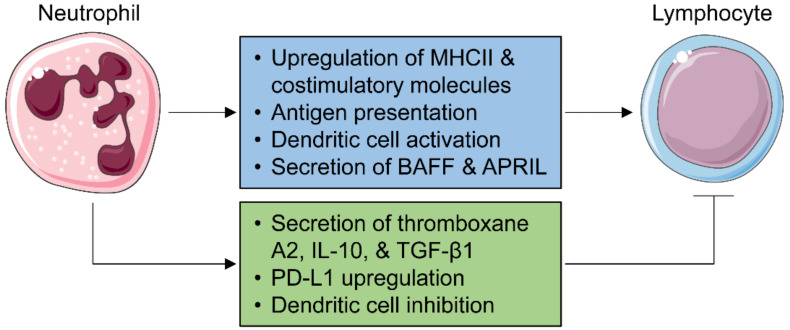
Neutrophil regulation of adaptive immunity. Following infection or inflammation, circulating neutrophils are recruited to lymph nodes, where they modulate the adaptive immune response. On the one hand, neutrophils are able to activate T cells and induce T cell proliferation and cytokine generation by upregulating major histocompatibility complex II (MHCII) and costimulatory molecules, presenting antigens, as well as activating dendritic cells. They also modulate B cell activation and survival by secreting B cell-activating factor (BAFF) and a proliferation-inducing ligand (APRIL). On the other hand, neutrophils can dampen adaptive immunity to prevent its overactivation by generating thromboxane A2, interleukin (IL)-10, transforming growth factor (TGF)-β1, upregulating programmed death ligand 1 (PD-L1), and inhibiting dendritic cell functions. Images of cells are from Servier Medical ART (Accessed date 1 June 2021 https://smart.servier.com).

Lymph node neutrophils may exert multiple functions, including pathogen killing, antigen transport, innate immune cell recruitment and removal, and regulation of an adaptive immune response [141]. Neutrophils exhibit temporary residency within the lymph node parenchyma and can act as sentinel cells to attract additional neutrophils in the event of bacterial dissemination to the lymph node [142]. Following ex vivo stimulation with an IgG immune complex, neutrophils upregulate the expression of major histocompatibility complex II (MHCII) and costimulatory molecules and increase T cell activation (Figure 3) [136]. In vivo, neutrophils are capable of delivering a circulating immune complex to lymph nodes, suggesting they can act as professional antigen-presenting cells [136,143,144]. Neutrophils activate DCs and enhance the subsequent T cell response [145,146]. Neutrophils induce T cell proliferation and cytokine production, supported by the finding that neutrophil depletion prevents T cell expansion in lymph nodes [139,147]. They also modulate B cell activation and survival by secreting B cell-activating factor (BAFF) [148] and a proliferation-inducing ligand (APRIL) [149]. 

On the contrary, neutrophils can also dampen adaptive immunity, serving as a negative feedback mechanism to prevent the overactivation of lymphocytes. Neutrophils suppress the T cell response by secreting thromboxane A2 [150], IL-10 [151], upregulating programmed death ligand 1 (PD-L1), and inhibiting DC functions (Figure 3) [147,152,153]. Lymph node neutrophils also dampen the humoral response in a TGF-β1-dependent manner [154]. Whether post-MI neutrophils enter heart-draining mediastinal lymph nodes and play essential roles in orchestrating an adaptive immune response is yet to be investigated. 

## 6. Anti-Neutrophil Strategies and Future Perspectives

Anti-neutrophil strategies include depletion of neutrophils, inhibition of neutrophil recruitment, blockade of neutrophil-derived deleterious mediators, and promotion of neutrophil clearance [155,156]. Depletion of neutrophils with the anti-Ly6G antibody has been extensively utilized in animal models [157]. This depletion, however, is only partially effective and transient, needing repetitive administration of anti-Ly6G antibodies. A recent study revealed that residual neutrophils, after anti-Ly6G treatment, are newly generated from the bone marrow, which have lower Ly6G expression and thus could escape anti-Ly6G-mediated depletion [158]. Furthermore, the authors developed a double antibody-based depletion strategy (anti-Ly6G plus anti-rat IgG) that achieves a more efficient, durable, and controlled reduction of neutrophils in vivo [158]. Neutrophil depletion can also be achieved by crossing MRP8-Cre mice with ROSA-iDTR^K1^ mice to generate PMN^DTR^ mice followed by diphtheria toxin treatment, or by crossing LysM-Cre mice with myeloid cell leukemia-1 (Mcl-1)^flox/flox^ mice followed by tamoxifen treatment [159]. Moreover, there are mouse models with constitutive neutropenia, including granulocyte colony-stimulating factor receptor (G-CSFR)^−/−^ and CXCR2^−/−^ mice [157]. Each model has its own limitations; please refer to the review by Stackowicz et al. for further details [157].

Although many experimental anti-neutrophil approaches have been shown to alleviate cardiac damage in the acute phase [10,160,161], the translation of this to the clinical scenario has not been successful. While the reasons are complex, one is our incomplete understanding of neutrophils’ multifaceted functions. In particular, the pro-reparative role of neutrophils should be taken into account when designing anti-inflammatory approaches. Any therapeutic strategies targeting neutrophils have to achieve a fine balance between the efficient reduction of pro-inflammatory roles and the preservation of the pro-reparative roles of neutrophils. For instance, one could combine the early administration of an agent that inhibits the pro-inflammatory response of neutrophils with the subsequent administration of an agent that activates the reparative response of neutrophils. In addition, the interaction of distinct immune cells with one another and with non-immune cardiac cells, such as cardiomyocytes, endothelial cells, and fibroblasts, further complicates the wound healing response after MI [162]. Therefore, this also needs to be taken into consideration when targeting immune cells, and further in-depth understanding of the underlying molecular mechanisms is required. 

## Data Availability

Not applicable.
